# Automated Inline Analysis of Myocardial Perfusion MRI with Deep Learning

**DOI:** 10.1148/ryai.2020200009

**Published:** 2020-10-21

**Authors:** Hui Xue, Rhodri H. Davies, Louise A. E. Brown, Kristopher D. Knott, Tushar Kotecha, Marianna Fontana, Sven Plein, James C. Moon, Peter Kellman

**Affiliations:** From the National Heart, Lung and Blood Institute, National Institutes of Health, 10 Center Dr, Bethesda, MD 20892 (H.X., P.K.); Barts Heart Centre, Barts Health NHS Trust, London, England (R.H.D., K.D.K., J.C.M.); Department of Biomedical Imaging Science, Leeds Institute of Cardiovascular and Metabolic Medicine, University of Leeds, Leeds, England (L.A.E.B., S.P.); and National Amyloidosis Centre, Royal Free Hospital, London, England (T.K., M.F.).

## Abstract

**Purpose:**

To develop a deep neural network–based computational workflow for inline myocardial perfusion analysis that automatically delineates the myocardium, which improves the clinical workflow and offers a “one-click” solution.

**Materials and Methods:**

In this retrospective study, consecutive adenosine stress and rest perfusion scans were acquired from three hospitals between October 1, 2018 and February 27, 2019. The training and validation set included 1825 perfusion series from 1034 patients (mean age, 60.6 years ± 14.2 [standard deviation]). The independent test set included 200 scans from 105 patients (mean age, 59.1 years ± 12.5). A convolutional neural network (CNN) model was trained to segment the left ventricular cavity, myocardium, and right ventricle by processing an incoming time series of perfusion images. Model outputs were compared with manual ground truth for accuracy of segmentation and flow measures derived on a global and per-sector basis with *t* test performed for statistical significance. The trained models were integrated onto MR scanners for effective inference.

**Results:**

The mean Dice ratio of automatic and manual segmentation was 0.93 ± 0.04. The CNN performed similarly to manual segmentation and flow measures for mean stress myocardial blood flow (MBF; 2.25 mL/min/g ± 0.59 vs 2.24 mL/min/g ± 0.59, *P* = .94) and mean rest MBF (1.08 mL/min/g ± 0.23 vs 1.07 mL/min/g ± 0.23, *P* = .83). The per-sector MBF values showed no difference between the CNN and manual assessment (*P* = .92). A central processing unit–based model inference on the MR scanner took less than 1 second for a typical perfusion scan of three slices.

**Conclusion:**

The described CNN was capable of cardiac perfusion mapping and integrated an automated inline implementation on the MR scanner, enabling one-click analysis and reporting in a manner comparable to manual assessment.

Published under a CC BY 4.0 license.

*Supplemental material is available for this article.*

**Summary**

The described convolutional neural network was capable of segmenting and determining the mean stress and rest myocardial blood flow in a manner comparable to manual segmentation.

**Key Points**

■ The study proposed and validated a convolutional neural network solution for cardiac perfusion mapping and integrated an automated inline implementation on the MR scanner, enabling one-click analysis and reporting.
■ The large training set included 1825 perfusion series from 1034 patients (mean age, 60.6 years ± 14.2), and the independent test set included 200 scans from 105 patients (mean age, 59.1 years ± 12.5).
■ Comparison of automated- and manual-derived myocardial blood flow measurement showed no differences on both global and per-sector basis (*P* > .80).

## Introduction

Myocardial perfusion MRI has proven to be an accurate, noninvasive imaging technique to detect ischemic heart disease (1). Quantitative MR perfusion is more objective (2), and automated in-line methods (3,4) offer improved efficiency of analysis. Compared with qualitative visual assessment, quantitative methods improve the detection of disease with a global reduction in flow as seen in balanced multivessel obstruction or microvascular disease (5).

Without automated segmentation of the MR perfusion maps, a reporting clinician would have to manually draw regions of interest to extract global or regional flow values. Objective perfusion assessment could be further facilitated by segmenting the myocardium to automatically generate the report, leading to a one-click solution to improve workflow. Automated MR perfusion measurement could serve as the input for downstream cardiovascular disease classification (6) in which pretrained convolutional neural network (CNN) models receive myocardial flow and other imaging features to predict the probability of ischemic heart disease. These previous studies used manual segmentation and can be automated with the proposed approach.

In this study, we proposed a deep CNN-based computational workflow for myocardial perfusion analysis using MRI. The right ventricular (RV) insertion points were determined to allow reporting of perfusion according to the standard 16-segment model proposed by the American Heart Association (AHA). To use the dynamic change of intensity that was the result of contrast material uptake, the proposed solution operates on the time series of two-dimensional (2D) perfusion images (referred to here as 2D+T) after respiratory motion correction. The performance of the trained CNNs was quantitatively evaluated by comparing against manually established ground truth for both segmentation accuracy and global as well as regional flow measures on an independent hold-out test dataset.

To promote the clinical validation and adoption of the proposed solution, the trained deep learning models were integrated onto MR scanners using the Gadgetron InlineAI toolbox (7). The CNN models were applied to the acquired images as part of the scanner computing workflow (inline processing) at the time of the scan, rather than as a part of postprocessing. The resulting segmentation results and analysis reports were available for immediate evaluation prior to the next image series. The method described here has been used in a prospective study of more than 1000 patients to demonstrate the prognostic significance of quantitative stress perfusion (8). A one-click solution to acquire free-breathing perfusion images, perform pixelwise flow mapping, and conduct automated analysis with a 16-segment AHA report generated on the MR scanner was demonstrated.

## Materials and Methods

### Imaging and Data Collection

In this retrospective study, the datasets consisted of adenosine stress and rest perfusion scans which were acquired at three hospitals (Barts Heart Centre, BHC; Royal Free Hospital, RFH; Leeds Teaching Hospitals, LTH) between October 1, 2018 and February 27, 2019. Data were acquired with the required ethical and/or audit secondary use approvals or guidelines (as per each center) that permitted retrospective analysis of anonymized data for the purpose of technical development, protocol optimization, and quality control. All data were anonymized and delinked for analysis by the National Institutes of Health with approval by the National Institutes of Health Office of Human Subjects Research (exemption #13156). The collected datasets were previously included in a recent study (9) that developed a left ventricular (LV) blood pool detection solution for arterial input function images, whereas this study used the datasets for perfusion myocardium segmentation.

A total of 1825 perfusion scans from 1034 patients (mean age, 60.6 years ± 14.2; 692 men) were assembled and split into training and validation sets used for CNN model training. An independent hold-out consecutive test set was assembled, consisting of 200 perfusion scans from 105 patients (mean age, 59.1 years ± 12.5; 76 men). The Table summarizes detailed dataset information. There was no overlap between the training and validation data to the independent test data (10). Among the assembled independent test data, 96 scans were acquired from 3-T scanners and 104 were from 1.5-T scanners.

**Table:**
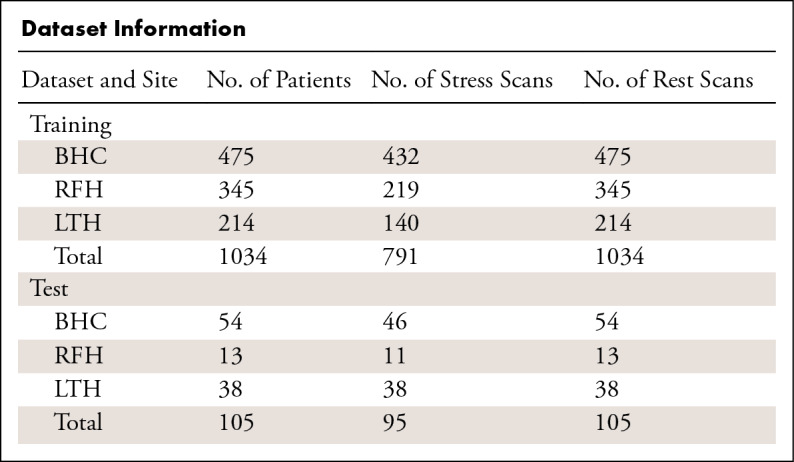
Dataset Information

### MRI Acquisition

Perfusion imaging used a previously published dual-sequence scheme (3). The imaging started after administering the contrast agent and with the acquisition of typical three 2D images cutting through the heart. This acquisition was repeated for every heartbeat to capture the contrast material passage. Details for MRI can be found in Appendix E1 (supplement).

### Data Preparation and Ground Truth Labeling

**Figure 1:**
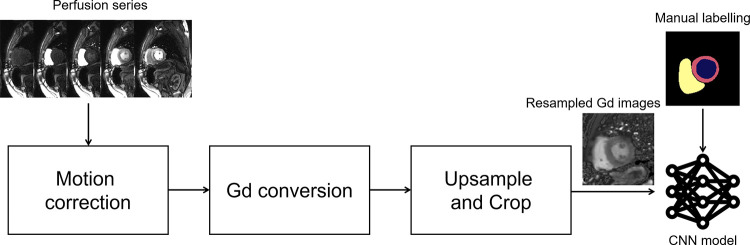
Data preparation for performing convolutional neural network (CNN)–based segmentation used in this study. Respiratory motion correction of perfusion images provides pixelwise alignment of myocardial tissue. Image intensities are corrected for surface coil inhomogeneity and converted to gadolinium (Gd) concentration units. Images are resampled to a fixed temporal and spatial resolution and cropped around the left ventricular cavity. The resulting 2D+T time series of images is input for CNN training, together with supplied manual labeling. 2D+T = two-dimensional perfusion images.

The perfusion image series underwent motion correction and surface coil inhomogeneity correction. Resulting images were spatially upsampled to 1.0 mm^2^, and the central field of view (176 × 176 mm^2^) was cropped. For the short-axis perfusion slices, the LV endo- and epicardial boundaries were manually traced, together with the RV (Fig 1). Information for data preparation and labeling can be found in Appendix E2 (supplement).

### Model and Training

**Figure 2:**
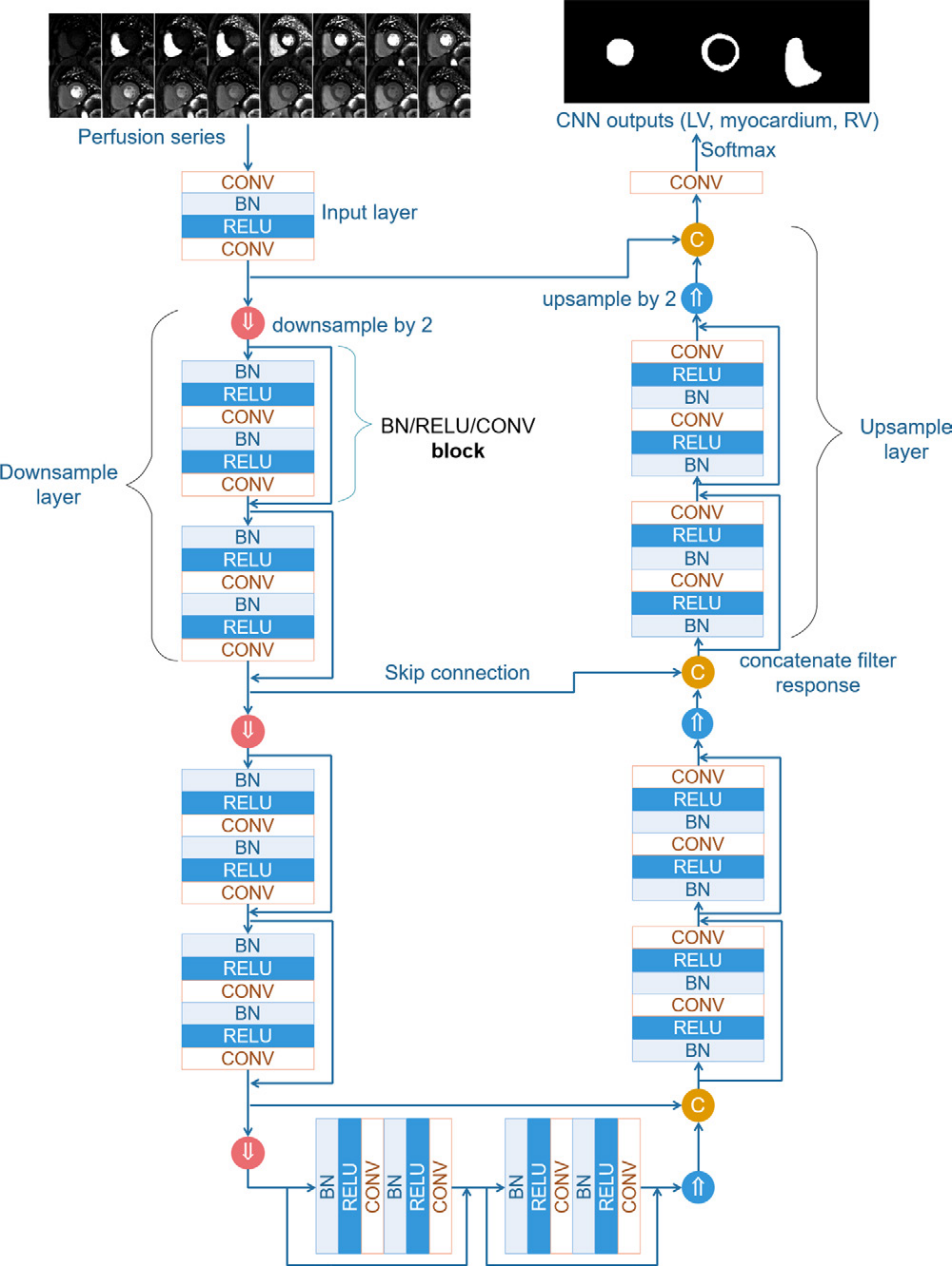
Schematic plot of the convolutional neural network (CNN) trained in this study. This network consists of downsampling and upsampling layers. Each layer includes a number of ResNet blocks. More layers and blocks can be inserted into the CNN to increase its depth. In the example illustration, two layers are used with two blocks for each layer. The total number of convolution blocks is 23. BN = batch normalization, C = concatenate filter response, CONV = convolution, LV = left ventricle, RELU = rectified linear units, RV = right ventricle.

The U-Net semantic segmentation architecture (11,12) was adopted for the perfusion segmentation. The neural net (Fig 2) consisted of downsampling and upsampling layers, each including a number of ResNet blocks (13) with batch normalization (14) and leaky rectified linear unit (15) nonlinearity. The data for training were split into a training set (87.5% of all studies) and a validation set (12.5% of all studies). The CNN model and optimization were implemented using PyTorch (16).

**Figure 3:**
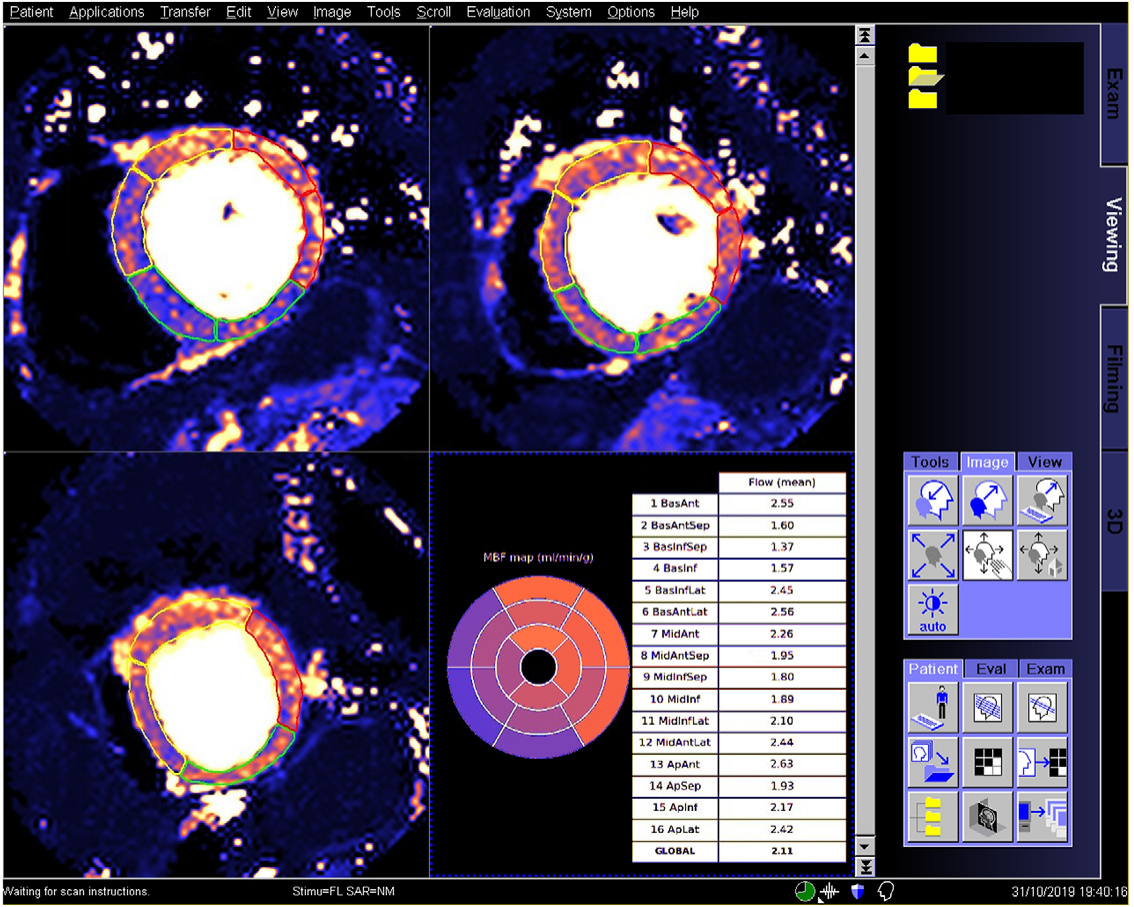
Screenshot in a patient undergoing an adenosine stress study demonstrates the proposed inline analysis solution on an MR scanner. Stress maps show regional flow reduction in septal and inferior sectors. The determined right ventricular insertion was used to split myocardium to American Heart Association (AHA) sectors, with the contours overlaid to mark territories. The inline reporting further produced a 16-sector AHA bulls-eye plot with global and per-sector flow measures reported in a table.

The trained model was integrated to run on MR scanners using the Gadgetron Inline AI (7) streaming software. A screenshot (Fig 3) illustrates the perfusion mapping with overlaid CNN-based segmentation and AHA report applied to a patient with reduced regional perfusion. This is a one-click solution for automated analysis of quantitative perfusion flow mapping.

Appendix E3 (supplement) provides details about model, training, and inline integration.

### Statistical Analysis

The segmentation of automated processing was compared with the manually labeled test set. Performance was quantified in both segmentation accuracy and myocardial flow measures. The Dice ratio for manual label and automatic segmentation masks was computed, together with the false-positive and false-negative errors. A false-positive error was defined as the percentage area of the segmented mask in the CNN result that was not labeled in the manual one. A false-negative error was defined as the percentage area of segmented mask in the manual that was not labeled in the automated result. The precision (defined as the percentage of segmented area in both the CNN and manual masks divided by CNN area) and recall (defined as the percentage of segmented area in both the CNN and manual masks divided by manual area) were also reported. The myocardium boundary errors (17), defined as the mean distance between myocardial borders of two masks, and the Hausdorff distance (18) were computed for the endo- and epicardium borders. The detection accuracy of RV insertion was measured by the angular difference between auto- and manual-determined direction vectors for RV insertion because only the orientation was needed for segmentation. Global and per-sector myocardial flow measures were used to quantify the CNN performance compared with manual results, displayed using Bland-Altman plots. In addition, contours were visually inspected for segmentation failures on all 200 test scans.

Results were presented as mean ± standard deviation. A *t* test was performed, and a *P* value less than .05 was considered statistically significant (Matlab R2017b; MathWorks, Natick, Mass). A *t* test was used to test whether there were significant differences on myocardial blood flow (MBF) values derived from manual and automated segmentation.

### CNN Data Sharing

To encourage researchers on other platforms to adopt the proposed solution, the CNN model files and other resources are shared openly (*https://github.com/xueh2/QPerf*).

## Results

### CNN Segmentation Overview and Optimization

An example of segmentation (Fig 4) illustrates the contours overlaid on perfusion images and corresponding flow maps. The trained CNN correctly delineated the LV cavity and myocardium. The RV insertion direction was accurately detected to allow sector division. The epicardial fat was correctly excluded from segmentation and the papillary muscles were avoided.

**Figure 4a:**
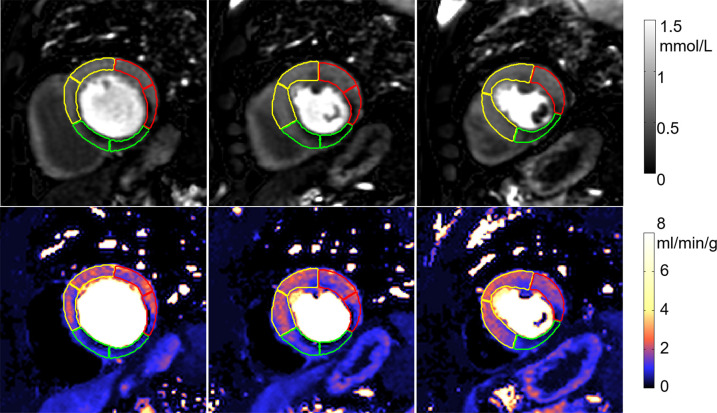
Adenosine stress perfusion images and myocardial blood flow (MBF) maps illustrate segmentation in the format of derived American Heart Association sector contours overlaid on flow maps. For each case, the first row is the images in gadolinium units and the second row is the MBF maps. Sector contours were overlaid to mark three territories for left anterior coronary artery (yellow), right coronary artery (green), and left circumflex (red). **(a)** Patient with single-vessel obstructive coronary artery disease in right coronary artery territory. Papillary muscle was not included in segmentation. **(b)** Patient with hypertrophic cardiomyopathy illustrates that the convolutional neural network–based segmentation works with thick myocardium and small cavity. The epicardial fat was correctly excluded.

**Figure 4b:**
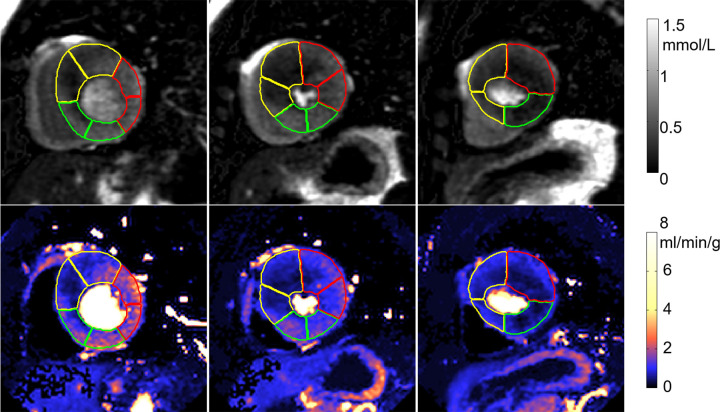
Adenosine stress perfusion images and myocardial blood flow (MBF) maps illustrate segmentation in the format of derived American Heart Association sector contours overlaid on flow maps. For each case, the first row is the images in gadolinium units and the second row is the MBF maps. Sector contours were overlaid to mark three territories for left anterior coronary artery (yellow), right coronary artery (green), and left circumflex (red). **(a)** Patient with single-vessel obstructive coronary artery disease in right coronary artery territory. Papillary muscle was not included in segmentation. **(b)** Patient with hypertrophic cardiomyopathy illustrates that the convolutional neural network–based segmentation works with thick myocardium and small cavity. The epicardial fat was correctly excluded.

### CNN and Ground Truth Segmentation Performance Comparisons

The mean Dice ratio of myocardium segmentation between CNN and manual ground truth was 0.93 ± 0.04 (90% confidence interval [CI]: 0.88, 0.97). False-positive and false-negative rates were 0.09 ± 0.06 (90% CI: 0.02, 0.18) and 0.06 ± 0.05 (90% CI: 0.005, 0.13). Precision and recall were 0.92 ± 0.06 (90% CI: 0.81, 0.97) and 0.94 ± 0.05 (90% CI: 0.87, 0.99). The myocardium boundary error was 0.33 mm ± 0.15 (90% CI: 0.13 mm, 0.52 mm). Given the training image spatial resolution of 1 mm^2^, the mean boundary error was less than 0.5 pixels. The mean bidirectional Hausdorff distance was 2.52 mm ± 1.08 (90% CI: 1.42 mm, 4.13 mm), and the mean angle between auto and manually determined RV insertion point directions was 2.65° ± 3.89 (90% CI: 0.28°, 5.95°).

The mean stress flow was 2.25 mL/min/g ± 0.59 for the CNN and 2.24 mL/min/g ± 0.59 for manual segmentation (*P* = .94). For rest scans, the CNN gave 1.08 mL/min/g ± 0.23 and manual measure gave 1.07 mL/min/g ± 0.23 (*P* = .83). The per-sector measures showed no difference between the CNN and manual measures (*P* = .92). Bland-Altman plots (Fig 5) compare automatic to manual processing of MBF for both global MBF and 16-sector values.

**Figure 5a:**
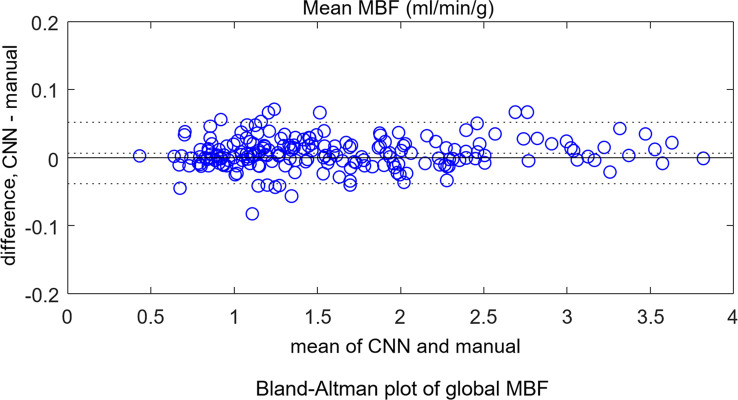
Bland-Altman plots for independent test dataset **(a)** global mean myocardial blood flow (MBF) and **(b)** per-sector measures. No significant differences were found between convolutional neural network (CNN)–derived results and manual measures. The dotted lines mark the 95% confidence range.

**Figure 5b:**
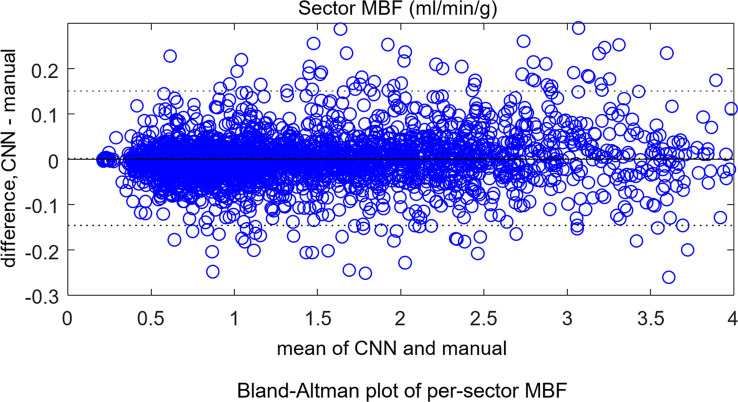
Bland-Altman plots for independent test dataset **(a)** global mean myocardial blood flow (MBF) and **(b)** per-sector measures. No significant differences were found between convolutional neural network (CNN)–derived results and manual measures. The dotted lines mark the 95% confidence range.

The performance was further evaluated separately for 3-T and 1.5-T test scans. The mean Dice ratio was 0.93 ± 0.04 for 3 T and 0.93 ± 0.03 for 1.5 T (*P* = .97). At 3 T, the mean stress flow was 2.20 mL/min/g ± 0.59 for CNN and 2.21 mL/min/g ± 0.59 for manual (*P* = .93). The mean rest flow was 1.08 mL/min/g ± 0.23 for CNN and 1.07 mL/min/g ± 0.23 for manual (*P* = .84). At 1.5 T, the mean stress flow was 2.29 mL/min/g ± 0.60 for the CNN and 2.29 mL/min/g ± 0.59 for manual (*P* = .97). The mean rest flow was 1.08 mL/min/g ± 0.23 for CNN and 1.07 mL/min/g ± 0.23 for manual (*P* = .93).

Contours were visually evaluated on all 200 test scans (three slices each). In a single stress case, one slice failed to properly segment the RV; however, the myocardium was properly segmented. A second rest case had one apical slice in which the myocardium segmentation included blood pool. There was apparent through-plane motion that was uncorrected. No other segmentation failures were found.

### CNN Speed Performance

The CNN model was integrated on the MR scanner. On Xeon Gold, model loading time was approximately 120 msec, and the applying model on the incoming perfusion series was approximately 250 msec per slice. For a typical three short-axis acquisition, inline analysis took less than 1 second on the central processing unit. On the older Xeon E5, model loading time was approximately 130 msec and applying models took 370 msec per slice.

## Discussion

This study presented a deep neural network–based workflow for automated myocardial segmentation and reporting of the AHA 16-sector model for pixelwise perfusion mapping. The derived myocardial measures were computed and reported inline on the MR scanner, taking just 1 additional second of inline processing time. Quantitative evaluation in this initial study demonstrated performance of myocardial segmentation and sector-based analysis that was well matched to a human expert. This study used stress and rest data from seven scanners at three sites at two field strengths, using more than 1800 consecutive scans for training and 200 for test. Bland-Altman analysis demonstrated a 95% CI for global MBF of 0.05 mmol/min/g compared with manual labeling, which was sufficient for automated detection and reporting. Prior work on segmenting perfusion (19) used much smaller datasets that resulted in much higher variance. A weighted sum loss function was used in this study and gave good accuracy. There are indeed many other alternatives, such as soft Dice ratio or focal loss (20), which can be effective in perfusion segmentation task. Which loss function is the best may vary for different applications. A comprehensive overview and implementation of many loss functions can be found at *https://github.com/JunMa11/SegLoss*.

The prognostic significance of proposed artificial intelligence application was studied and recently published in Knott et al (8). In this study, 1049 patients with known or suspected coronary artery disease underwent stress MR perfusion scans and were analyzed with the proposed CNN models, showing reduced MBF and myocardial perfusion reserve measured automatically using artificial intelligence quantification of cardiac MR perfusion (2). This study demonstrates the relevance of automated myocardial segmentation in cardiac MR stress perfusion.

Automated cardiac MR image analysis has been attempted over a long period of time (21). Most work focused on cine image analysis (eg, MICCAI 2017 ACDC challenge [22]). The first deep learning study, which was based on a large data cohort and reported performance matching human level, was published in 2018 (23). Since then, deep neural nets were applied to other cardiac MR imaging applications, such as T1 mapping and cardiac late enhancement segmentation (24). Our approach used the temporal information through the whole bolus passage (2D+T) to exploit the contrast dynamics for detecting both RV and LV which enabled finding the RV insertion point. The epicardial fat, showing no dynamic intensity changes, was correctly excluded from segmentation which would have been more difficult to avoid on a single static image.

This study had several limitations. First, the presented study was conducted on MR scanners from a single vendor. Although the specific imaging dual sequence used may not be available on other platforms, the proposed segmentation method and CNN models may be still applicable. Second, although the proposed algorithm works well for most cases, a few cases are challenging. For example, in the case of severe hypertrophy, some slices may not exhibit any blood pool (ie, complete extinction) where no endocardial contours are drawn. Third, the CNN models are currently trained for short-axis slices and cannot be applied to long-axis views. New training and test datasets are needed to extend segmentation to long-axis slices. In the case where the basal slice may cover some portion of the outflow tract, the proposed algorithm will avoid the blood pool and divide a segment accordingly or may skip a segment entirely, resulting in incomplete segmentation. Fourth, in cases of severe respiratory motion that is beyond the capacity of the in-plane retrospective methods, portions of the myocardium may be blurred, where CNN segmentation can perform poorly. However, in these cases manual segmentation is difficult as well.

Another limitation of this study was the single operator for data labeling. The interoperator reliability was not tested in this article. Because this solution had been deployed to MR scanners, clinical collaborators have started to use this solution (8,25), but more clinical validation is required to further validate this solution.

In conclusion, we demonstrated automated analysis can be achieved on clinical scanners for perfusion MRI. Deep learning enabled inline analysis immediately after data acquisition as part of imaging computation, therefore making the process more objective, convenient, and faster, reducing clinical burden.

## APPENDIX

Appendices E1-E3 (PDF)
