# Supplementary material for: Automated Inline Analysis of Myocardial Perfusion MRI with Deep Learning
Source: Radiol Artif Intell. 2020 Oct 21;2(6):e200009. doi: 10.1148/ryai.2020200009 (PMC7706884; doi:10.1148/ryai.2020200009)
Supplement: Appendices E1-E3 (PDF) [file ryai200009suppa1.pdf]

©RSNA, 2020
10.1148/ryai.2020200009

Appendix E1

MRI Acquisition

Perfusion imaging used a previously published dual-sequence scheme (3). A low-resolution arterial input function imaging module was inserted before the perfusion imaging and performed after the *R*-wave with short delay time. Typical parameters for myocardial imaging: FOV 360 × 270 mm^2, slice thickness 8 mm, imaging matrix 192 × 111, interleaved acceleration R = 3, TE = 1.04 ms, TR = 2.5 ms, TD = 40 ms, flip angle 50°, FISP readout. Gadolinium [Gd] contrast agent (XX and XH: gadoterate meglumine, Dotarem; Guerbet, Paris, France; LTHT: Gadovist, Leverkusen, Germany) was administered as a bolus of 0.05 mmol/kg at 4 mL/sec with 20 mL saline flush using power injectors (Medrad MRXperion Injection System, Bayer). For stress perfusion, adenosine was administered by continuous intravenous infusion for 4 min at a dose of 140 μg/kg/min before contrast injection (increased to 175 μg/kg/min for a further 2 minutes based on patient's response). The imaging started by acquiring three proton density weighted images, followed by saturation recovery images. Every perfusion image was acquired as a 2D image cutting through the heart and this acquisition was repeated for every heart beat to capture the contrast passage, typically lasting 60 heart beats. This resulted in the 2D+T time series where images were acquired consecutively in time. Details of imaging and perfusion mapping can be found in Kellman et al (3). Datasets were acquired using both 1.5 T (four MAGNETOM Aera, Siemens AG Healthcare, Erlangen, Germany) and 3 T (three MAGNETOM Prisma, Siemens AG Healthcare) MR scanners.

Appendix E2

Data Preparation and Labeling

Perfusion image series underwent motion and surface coil inhomogeneity correction. Motion correction utilized nonrigid image registration in an iterative manner. To compensate for substantial image contrast variation during the contrast bolus passage, instead of directly registering perfusion images against each other, synthetic perfusion series were derived from a Karhunen-Loève transform. Motion correction was achieved by registering perfusion images pairwise with the synthetic series. The detailed algorithm was presented in Xue et al (4). After correcting respiratory motion, surface coil inhomogeneity was corrected using the proton density images and the normalized intensities were converted to gadolinium concentration units (mmol/L) (3). To compensate for heart rate variation and mis-triggering, the perfusion series was temporally resampled using linear interpolation which also compensated for possible missed triggers. This interpolation resulted in a fixed sampling corresponding to a heart rate of 120 bpm. The temporal resampling step did not lead to spatial blurring since it was performed after motion correction.

Since the gadolinium concentration series was corrected for signal nonlinearity and surface coil inhomogeneity, it had the benefit of reducing the dynamic range and providing a fixed signal range for neural nets, compared with perfusion intensity images. This image series was spatially upsampled to 1.0 mm^2 spatial resolution and the central field of view (176 × 176

mm^2) was cropped. The left ventricular (LV) blood pool was detected from the arterial input function series which was imaged at the basal plane at diastole. The location of the LV blood pool from this step was used to center the cropped image (9). For the short axis perfusion slices, the LV endo-and epicardial boundaries were manually traced, together with the right ventricle (RV) (Fig 1). The RV insertion point was determined from the segmented right ventricular and LV center as the rightmost pixel. The training and test datasets were carefully labeled by one operator (10 years of experience in perfusion imaging).

Appendix E3

Model, Training and Inline Integration

Details of neural net model.—

The first 48 images were empirically selected, starting at the first saturation recovery image. This resulted in an image array of 176 × 176 × 48 per section, covering the first-pass bolus passage of injected contrast agent. A total of 262800 2D images were then used for training the neural networks. The U-net semantic segmentation architecture (11,12) was adopted for the perfusion segmentation. The neural net (Fig 2) consisted of downsampling and upsampling layers, each including a number of ResNet blocks (13). The downsampling and upsampling operations were inserted between layers to change the spatial resolution. For simplicity, two convolutional layer operations with the same number of output filters were added to each block, together with Batch Normalization (14) and LeakyRelu (15) nonlinearity. All convolutional layers used a 3 × 3 kernel with stride of 1 and padding of 1. Following the principle of U-net, the downsampling and upsampling layers were connected with Skip-connections. The spatial resolution was reduced by going through the down-sampling branch with the number of convolution filters increased. The up-sampling branch increased the spatial resolution and reduced the number of filters. The network was able to learn features from this coarse-to-fine pyramid thereby selecting an optimal filter combination to minimize the loss function.

The final convolutional layer output was a 176 × 176 × 3 array of scores representing segmented classes, which were converted to probability through a softmax operation. Establishing the anatomic context of LV cavity, myocardium, and right ventricle was facilitated by using a single trained CNN. The loss function was a weighted sum of cross-entropy and the intersection over union. This cost function optimizes the overlap between the detected mask and ground-truth while maximizing the probability for a pixel to be correctly classified, previously shown to improve segmentation accuracy (26). The trained CNN models were applied to both stress and rest test scans.

Training and hyperparameter search.—

The data for training was split into a training set (87.5% of all studies) and a validation set (12.5% of all studies) and the CNN model and optimization was implemented using PyTorch (16). Training was performed on a Linux PC (Ubuntu 18.04) with four NVIDIA GTX 2080Ti GPU cards. ADAM optimization was used with initial learning rate equal to 0.001 (beta = 0.9 and 0.999; epsilon = $1 \cdot 10^{-8}$). Learning rate was reduced by a factor of 2 for every 10 epochs. Training took 60 epochs and best model was selected as the one giving best performance on the validation set.

A hyperparameter search was conducted to test different network parameter combinations (2 to 4 resolution layers, 2 to 4 blocks per layer, and number of convolution filters of either 64, 128 and 256). After the hyperparameter search, best performance was found for an architecture containing two down-sampling and up-sampling layers, with two ResNet blocks for the first layer and three blocks for the second. This led to a deep net with 23 convolution layers in total. On the tested hardware, training took approximately 8 hours for 60 epochs.

Inline integration of trained models.—

The trained model was integrated to run on MR scanners using the Gadgetron Inline AI (7) streaming software which provides flexible interfaces to load pretrained neural networks and apply them on incoming new data. This involved transferring model objects from Pytorch to C++ and passed data from C++ to Pytorch modules. Model inference was chosen to utilize a central processing unit (CPU) which was sufficiently fast for clinical usage.

Perfusion segmentation functionality was performed after inline perfusion mapping. As soon as a perfusion scan was configured, the pretrained model was loaded into the Gadgetron runtime environment. Following image reconstruction and preprocessing, models were applied to the incoming 2D+T image series for each slice. Resulting segmentation was used to generate the 16-sector measurement of perfusion and produce a summary report. All steps were fully automatic without any user interaction. A screenshot (Fig 3) illustrates the perfusion mapping with overlaid CNN based segmentation and AHA report, applied to a patient with reduced regional perfusion. This is a "one-click" solution for automated analysis of quantitative perfusion flow mapping. Trained models were tested on two computing servers for timing. Xeon Gold: 2×Intel Xeon Gold 6152 CPU @ 2.1GHz, released in 2017, 192 GB RAM. Xeon E5: Intel Xeon E5–2680 CPU, released in 2012 and 64G RAM.

Availability of Data and Material

The raw data that support the findings of this study are available from the corresponding author upon reasonable request subject to restriction on use by the Office of Human Subjects Research. The source file to train the CNN model and example datasets are shared at https://github.com/xueh2/QPerf.git.

Reference

26. Milletari F, Navab N, Ahmadi SA. V-Net: Fully convolutional neural networks for volumetric medical image segmentation. 4th Int Conf 3D Vis, 2016; 565–571.
